# Early Humans’ Egalitarian Politics: Runaway Synergistic Competition under an Adapted Veil of Ignorance

**DOI:** 10.1007/s12110-014-9203-6

**Published:** 2014-07-05

**Authors:** Marc Harvey

**Affiliations:** 18 St-Cyrille, Montréal, QC Canada H2V 1H8

**Keywords:** Human uniqueness, Balanced deterrence, Political egalitarianism, Obligate team synergy, Synergistic competition, Low-profile prestige, Political niche construction, Egalitarian culture and communications, Immaterial commons

## Abstract

This paper proposes a model of human uniqueness based on an unusual distinction between two contrasted kinds of political competition and political status: (1) *antagonistic competition,* in quest of dominance (antagonistic status), a zero-sum, self-limiting game whose stake—who takes what, when, how—summarizes a classical definition of politics (Lasswell 1936), and (2) *synergistic competition,* in quest of merit (synergistic status), a positive-sum, self-reinforcing game whose stake becomes “who brings what to a team’s common good.” In this view, Rawls’s (1971) famous virtual “veil of ignorance” mainly conceals politics’ antagonistic stakes so as to devise the principles of a just, egalitarian society, yet without providing any means to enforce these ideals (Sen 2009). Instead, this paper proposes that human uniqueness flourished under a real “adapted veil of ignorance” concealing the steady inflation of synergistic politics which resulted from early humans’ sturdy egalitarianism. This proposition divides into four parts: (1) early humans first stumbled on a purely cultural means to enforce a unique kind of within-team antagonistic equality—dyadic balanced deterrence thanks to handheld weapons (Chapais 2008); (2) this cultural innovation is thus closely tied to humans’ darkest side, but it also launched the cumulative evolution of humans’ brightest qualities—egalitarian team synergy and solidarity, together with the associated synergistic intelligence, culture, and communications; (3) *runaway synergistic competition for differential merit among antagonistically equal obligate teammates* is the single politically selective mechanism behind the cumulative evolution of all these brighter qualities, but numerous factors to be clarified here conceal this mighty evolutionary driver; (4) this veil of ignorance persists today, which explains why humans’ unique prosocial capacities are still not clearly understood by science. The purpose of this paper is to start lifting this now-ill-adapted veil of ignorance, thus uncovering the tight functional relations between egalitarian team solidarity and the evolution of human uniqueness.

This paper proposes an “egalitarian hypothesis of human uniqueness” which clarifies and reconciles a wealth of extant cross-disciplinary evidence, from primatology and human paleontology to hunter-gatherers’ behavioral ecology, anthropology, and archaeology, including general principles of animal behavior, evolutionary psychology, and political philosophy. The model also emphasizes many political aspects of the human condition—notably, *the quest for maximum merit (“like” scores) and minimum demerit (“dislike” scores) among peers and teammates*—which, while extremely familiar, remain neglected in behavioral ecology (but see Henrich and Gil-White 2001:170) and very confused in political economics. For example, citizens of modern meritocracies are familiar with the idea that individual differences in merit—as roughly measured by “*who brings what* to the common good,” a synergistic stake therefore—are often rewarded by large disparities in “*who gets what share* of the common output” (see Piketty and Saez 2014), an antagonistic stake according to the simple definition proposed here. This conventional wisdom holds that such contrasted antagonistic rewards are powerful individual incentives without which the common good of economic and cultural progress would remain sluggish.

By contrast, this paper proposes that, starting from chimpanzeelike societies which were far from egalitarian, the evolutionary and cultural progress which gave rise to human uniqueness derived from the steady inflation of a purely synergistic form of competition—a race to maximize merit (“like” scores) and minimize demerit (“dislike” scores)—wherein everyone got an equal share of team synergy’s common benefits, and this, among other reasons, because *any unequal share was associated with strong “dislike” scores (obvious signs of demerit) liable to all sorts of collective punishment.* This kind of coalitionary punishment is often invoked to explain human egalitarianism, as recently emphasized in Boehm’s (2012) landmark *Moral Origins*. However, *within an already hierarchical order, any coalition will necessarily end up hierarchical too,* eventually changing the political order, but only to another hierarchical one. Therefore, the capacity to collectively weed out and punish individual demerit is certainly an important aspect of human egalitarianism, but it cannot be its primary cause, nor the primary driver of human uniqueness’s brighter aspects.

The triggering of early humans’ unique political egalitarianism, and its maintenance up until its Neolithic breakdown, is here more precisely ascribed to a simple, fast-spreading cultural innovation: the antagonistic balance of deterrence afforded by handheld weapons—clubs, sticks, and stones, or “technological weapons” as proposed by Chapais (2008:176–177)—leading to a purely dyadic form of antagonistic equality, which nonetheless ensures that no one can impose his will but through a process of *quorum formation,* 1 the ensuing synergistic quorums necessarily remaining egalitarian for the same reason. In this view, collective punishment becomes a challenge of quorum formation among antagonistic equals, but so do all other, more positive collective endeavors. Hence the notion of egalitarian team synergy, with the ensuing *selective yet quorum-dependent political competition* among obligate teammates with different political aptitudes, attitudes, and behaviors, both antagonistic and synergistic. Now, even if the number of possible teammates is limited within a small egalitarian team, all their appreciated, merit-worthy attitudes become *open-access behaviors,* 2 which can be freely emulated and followed, and whose possible combinations (the different “what, where, when, how, and with whom” of collective action) multiply accordingly, hence the inflationary potential of synergistic competition among antagonistic equals.

Why compete for maximum merit (“like” scores) and minimum demerit (“dislike” scores) in such uniquely egalitarian settings? This question will be examined repeatedly throughout this paper, but the shortest and most proximate answer boils down to the fact that, among antagonistic equals, differential merit (synergistic political fitness) becomes the best means to recruit and be recruited—that is, the best means to build up or join the most effective synergistic quorums according to one’s own best interests, merit also remaining the best means to avoid quorum punishment. If correct, this suggests that the root of humans’ brightest qualities, the *open-access, quorum-dependent quest for merit among antagonistically equal obligate teammates,* is somewhat neglected by current evolutionary approaches of animal and human behavior. The contrast with the attention granted to limited-access dominance is striking. According to one of this paper’s main propositions, this disregard for open-access merit is a predictable effect of the adapted veil of ignorance which has concealed synergistic competition’s mighty dynamics since the onset of human egalitarianism. The ensuing paradoxical mix of procedural familiarity and foggy declarative awareness persists today, thus demanding that the veil in question be lifted at least somewhat before discussing human uniqueness’s other aspects, especially the darker ones. Indeed, without a proper lens to see the light first, it is extremely difficult to imagine how the darkness of balanced deterrence could have triggered and sustained the inflation of open-access quorum synergy and the evolution of all the brighter aspects of human uniqueness all along the Paleolithic.

This paper thus proceeds in two parts, a first theoretical half on egalitarian team synergy’s quorum-dependent dynamics with its adapted veil of ignorance, and a second half on early humans’ unique behavioral ecology as revealed behind this veil. Likewise for the abundant empirical evidence on hunter-gatherers’ egalitarianism, as for Rawls’ virtual veil of ignorance: both are discussed in the second half because their peculiarities are better appreciated after some examination of egalitarian team synergy’s dynamics. Current models of cooperation and human uniqueness are discussed alongside. Concerning the antagonistic equality afforded by handheld weapons, recall the famous quote from Lee (1969) reported by Trivers (1971:45) about a common saying of Bushmen when their discussions near confrontation: “We are none of us big, and others small; we are all men and we can fight: I’m going to get my arrows.” The similar leveling effect of more primitive weapons is discussed in this paper’s second half.

## Mapping Political Synergy’s Territory

The first half of this paper divides into three parts, a first one mapping political synergy’s territory, a second one comparing antagonistic competition and synergistic competition, to see how and where cumulative inflation develops once team synergy arises among antagonistic equals, and a third one comparing the numerous stark contrasts between dominance and merit among antagonistic equals, to see why an adapted veil of ignorance necessarily develops over the runaway quest for the latter. To begin, I briefly outline the unmapped territory of synergistic politics, synergistic competition and teammate choice in social animals so as to picture the quest for merit’s broader biological context. Of course, the notion of synergy (from the Greek roots *syn* and *ergon,* for “work together”) overlaps that of cooperation, but the former better emphasizes the unavoidably competitive and politically selective nature of cooperation in social animals as soon as they have enough time to know each other and thus build up sturdy (antagonistic and synergistic) reputations for themselves; hence the political—who’s who—nature of their interactions. Three branching pairs of core distinctions can briefly delineate the field of synergistic politics:

1. A first already evoked distinction between two opposite forms of political competition:
*Antagonistic* competition, about *who gets what share* of the available material resources, whether individually gathered or collectively produced.
*Synergistic* competition, about *who brings what* contribution to the common good, that of a team or any other dyadic or polyadic partnership among familiar individuals.
2. A second distinction between two branching forms of individualized within-group synergy, beyond the anonymous forms of herd synergy (Hamilton 1971):
*Micro synergy:* mutualism, reciprocity, and cooperation among a limited number of familiar, well-identified group mates, usually no more than two or three.
*Whole-group team synergy* in between-team competition.
3. A third distinction between two branching kinds of whole-group team synergy:
*Hierarchical* team synergy: whole-group teamwork under a stable antagonistic order.
*Egalitarian* team synergy: whole-group teamwork among antagonistically equal teammates—a human singularity, as proposed here.

According to this framework, human uniqueness developed because early humans were the first social animals to cross the threshold of “egalitarian team synergy” from a previous state of chimpanzeelike “hierarchical team synergy.” In this view, demanding forms of whole-group cooperation and collective action such as teamwork and team solidarity in between-team competition cannot normally develop except within an already stable and reliable antagonistic rank order, thus usually giving rise to strictly hierarchical forms of whole-group team synergy. In such circumstances typical of most cooperative breeding animals (a wolf pack, for example), whole-group team synergy remains a strictly *limited-access game* where (a) positive-sum synergistic displays of prestige can sometimes replace mere zero-sum antagonistic displays of dominance (Zahavi 1977, 1990, 1995; Zahavi and Zahavi 1997), but where (b) leadership and most of team synergy’s positive-sum benefits are monopolized by alpha dominants because of the underlying hierarchical order. Early humans escaped this political straitjacket thanks to the antagonistic balance of deterrence afforded by handheld weapons. Which suggests that, for animals already specialized in the mutual dependence and the tightly constrained forms of synergistic competition typical of hierarchical teams, such a fast neutralization of within-team antagonistic competition could have led to an inflationary escalation of synergistic competition: an open-access runaway quest for “like” scores and merit-worthy displays through an escalatory “who brings what, when, how” to a team’s common good. Among antagonistic equals, this runaway expansion of synergistic competition would thus become an unbridled evolutionary driver responsible for the emergence and diversification of all the brighter aspects of human uniqueness, notably the inflationary expansion of synergistic intelligence, culture, and communications (language).

## Equality’s Perils and Promises

Of course, this brighter dynamics of open-access synergy is far from a guarantee of political stability or collective progress. Indeed, while the number of possibly effective quorums always remains limited, in contrast, an open access to merit greatly increases the number of possible, and sometimes opposing, proposals about quorum synergy’s “what, where, when, how, and with whom?” with ensuing, unavoidably numerous and predictable proto-debates and negotiations, often confronting the merit of different proposers. As a result of such *open-access daily deliberations for quorum formation,* egalitarian team synergy becomes uniquely rich in possibilities for both (a) progresses in synergistic coordination, effectiveness, and solidarity but also (b) regresses and stagnations because of collective mistakes, fruitless oppositions, conflicts, and scissions. The bio-physical world is complex, often obscure and unpredictable, and collectively tackling its innumerable challenges is often difficult, even for good-willed contemporary humans enlightened by science. But remarkably, *there is one debate-related domain in which cumulative inflation is not only possible but almost unavoidable, once open-access antagonistic equality is ensured among obligate teammates: this is the limitless immaterial world of low-cost synergistic communications, the realm of synergistic coordination signals, which immediately benefit all those who produce and catch as many intelligible messages as possible while involved in the open-access synergistic debates required for quorum formation.* Such coordination signals don’t have to be costly to be reliable (Grafen 1990; Zahavi 1977) because they are exchanged between familiar teammates trying to understand and influence each other while managing their egalitarian teamwork. For the same reason, these low-cost signals can be simple, arbitrary—thus symbolic and infinitely modifiable—utterances or signs, provided they are understood by their intended audience, whose attention and reactions will often depend on the senders’ merit. Boesch (1991) reports a unique instance of such simple symbolic signals in wild chimpanzees. No wonder that egalitarian early humans could have greatly improved such a trade, the quest for individual merit thus ultimately fueling the daily proximate expansion (production and imitation) of the most merit-worthy symbolic signals. The ensuing domain-specific yet predictably limitless informational inflation is the topic of the next section, focused on the stark differences therein between limited-access hierarchical and open-access egalitarian synergistic debates.

## Egalitarian Communications’ Inflation

As evoked above, the difference between antagonistic and synergistic competition boils down to the fact that the first is a *zero-sum* (often even negative-sum), *self-limiting* game whereas the second is the opposite, a *positive-sum, self-reinforcing* one. Antagonistic competition is zero-sum because what is taken by the winners is lost for the losers, and it is also self-limiting because animals necessarily avoid useless fights and fruitless interactions and debates, thus converging toward antagonistic equilibriums, those of linear rank orders. Conversely, synergy is a positive-sum self-reinforcing game because it favors the recurrence and multiplication of mutually fruitful interactions, hence a possible divergence from a status quo. Of course, this positive-sum self-reinforcing effect can develop even within a hierarchy, but it cannot escape the straitjacket of such a rank order. This is precisely where the neutralization of antagonistic competition, thanks to balanced deterrence, produces its inflationary effects.

The political straitjacket constraining social animals’ synergy is examined first. Indeed, synergistic competition is severely bounded by these animals’ hierarchical orders for three reasons. Briefly:

1. Because, except for their closest beta opponents, alpha dominants can easily oppose any cooperative association among their lower subordinates, especially if this cooperation jeopardizes their dominance.
2. Because even for politically inconsequential forms of micro synergy (technical cooperation among a limited number of participants), subordinate cooperators can hardly defend the material products of their cooperation (food reserves, for example) against the plundering of dominants, a permanent risk much more detrimental to the development of cooperation than cheating and free-riding, two strategies also hardly sustainable within small groupings of familiar individuals.
3. Finally, when dominants welcome cooperation as they do in cooperative breeders—among whom some subordinate helpers find some benefits in joining such dominant-managed cooperation, most often between parents and offspring—the ensuing tightly *hierarchical team synergy* has three unavoidable properties constitutive of a genuine political straitjacket:
  - The participants and their synergistic interactions remain closely policed by alpha leaders.
  - Such hierarchical team synergy therefore remains strongly biased in favor of alpha breeders.
  - The quest for (synergistic) prestige can possibly develop as a useful daily substitute for (antagonistic) dominance (Zahavi 1977, 1990), but for subordinate helpers, this kind of limited-access competition remains bounded between two evils: (i) the narrow benefits afforded by the hierarchical order and the alpha breeders’ ruthlessly enforced reproductive privileges, and (ii) the perilous option of leaving the group and starting a new political career elsewhere, when possible.

The contrast with early humans’ synergistic competition among antagonistic equals is striking. Indeed, here is a competitive quest for open-access merit which offers much more leeway to all contestants whatever their physical strength, thus favoring the exploitation of all sorts of other individual qualities beyond mere antagonistic capacity. But in addition, it is a competition in which one of the best ways to succeed is to contribute more to the welfare of one’s competitors, provided they belong to the same party. All of synergy’s material outputs (food, shelter, etc.) being then shared equally (see the discussion of hunter-gatherers’ egalitarianism below), and the winners being those who bring the most to the welfare of others, there are much fewer reasons to stop such contests and reach a stable equilibrium. Of course, as mentioned above, crucial factors still hinder such open-access cooperation, but remarkably, they are not the ones usually considered in game-theoretical models—cheating and free-riding (Axelrod and Hamilton 1981; Trivers 1971). Again, once the numerous “what, where, when, how, and with whom” of quorum synergy become open-access, their possible combinations multiply accordingly, while the best course of collective action is rarely evident, even for contemporary humans. Hence the combinatorial explosion of debates and deliberations, then as now, however limited the means of deliberation at the onset. A totally new game-theoretical question therefore arises: what happens to cooperation once cheating and free-riding are barely possible because of familiarity and quorum punishment and, to the contrary, competitive synergy is the most effective means to serve both one’s own best interests and those of the whole community? Progress is not guaranteed, but another consequence of open-access merit is: *whatever consensus develops, it will be emulated, followed, and eventually reproduced across generations because all associated behaviors become merit-worthy in the eyes of consensus members while remaining open-access thanks to antagonistic equality*.

**Fig. 1:**
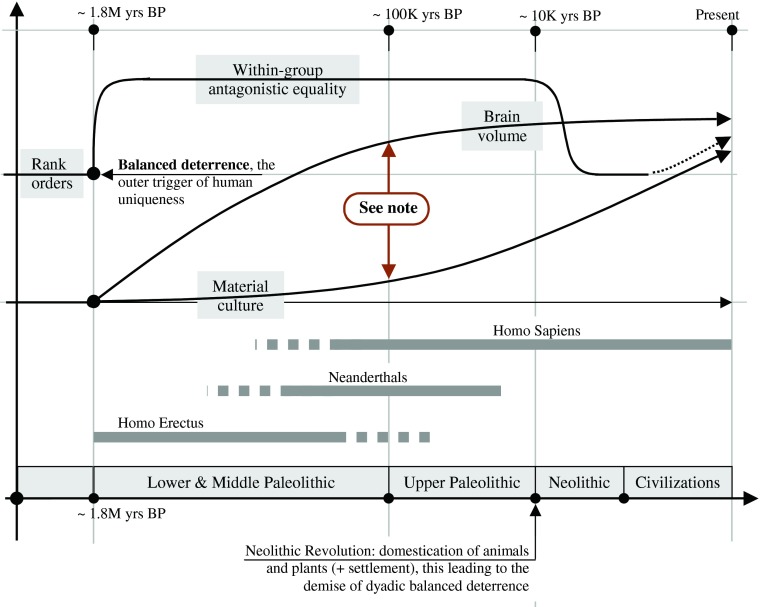
A schematic timeline showing human evolution’s main features and their starkly asymmetrical developments. The paleontological record on *Homo erectus* clearly features an unprecedented mix of geographical expansion together with a steady expansion of the brain volume, yet without any associated sign of significant cultural progress all along the Lower and Middle Paleolithic. Indeed, after reviewing the available evidence on the very slow developments in stone tool technologies during this whole period, Mithen (1999:394) concludes: “As several archeologists have commented, it is the remarkable stability in technology, and culture in general, during the Middle Pleistocene which is in most need of explanation, not the presence of change.” Boaz and Ciochon (2004:141) concur: “We agree that some sort of cognitive advance had occurred in *Homo erectus*. But equally intriguing is the fact that whatever that advance represented, it seems to have stayed virtually static for some one million years, the length of time that bifaces and similar chopping tools dominated *Homo erectus* lithic culture.”

In this way, the quest for open-access merit can foster the improvement of any already-established equilibrium, provided these improvements fit the preferences of enough teammates. Yet, once confronted by the biophysical world’s complexities, obscurities, and uncertainties, this political freedom can also lead to all sorts of costly mistakes; hence the adapted conservatism and relatively slow progress of early humans’ material culture evidenced by the archaeological record. Yet again, there is one *unbounded immaterial world,* that of open-access synergistic communications, with its politically selected means of debating and understanding, where limitless cross-generational inflation is guaranteed from the outset of egalitarianism, driving the steady expansion of humans’ linguistic capacities with the associated, fossilizable, steadily increasing brain volume, another salient feature of humans’ paleontological record—see Fig. 1’s schematic timeline, to be discussed further below.

## Political Selection’s Devo-Evo Mechanics

A derived, detailed model of language evolution will be proposed in a subsequent article, but for now, a simple evolutionary framework already emerges from the above basic dynamics: whatever the contrasted paces of early humans’ progresses in material and symbolic culture, the cumulative growth of human uniqueness stems from *a multilevel, bottom-up, devo-evo* (developmental first, and evolutionary next; see Hall 2000) *process of political selection* where (a) the quest for merit drives the “devo” arm of the process, the steady cross-generational growth of synergistic competition’s pressures bearing on all individuals, and (b) another reproductively selective mechanism drives the “evo” arm of the process. In short, starting with the devo arm:

1. Individual political actors select their individual and collective behaviors, choose their teammates, and elect their leaders according to their differential merit in all their daily activities—partners, quorums, and party choices—throughout their lifetime.
2. These lifelong synergistic selections of partners, leaders, behaviors, and signals thus simultaneously select the most effective and appreciated cultural ways and means of political and technical teamwork, the most appreciated “what, when, where, and how” of synergistic (who’s who) competition. These open-access behaviors therefore become ever more numerous, demanding, yet requisite means to acquire merit, thereby increasing the “devo” pressures of political selection and synergistic competition on all participants.

**Fig. 2:**
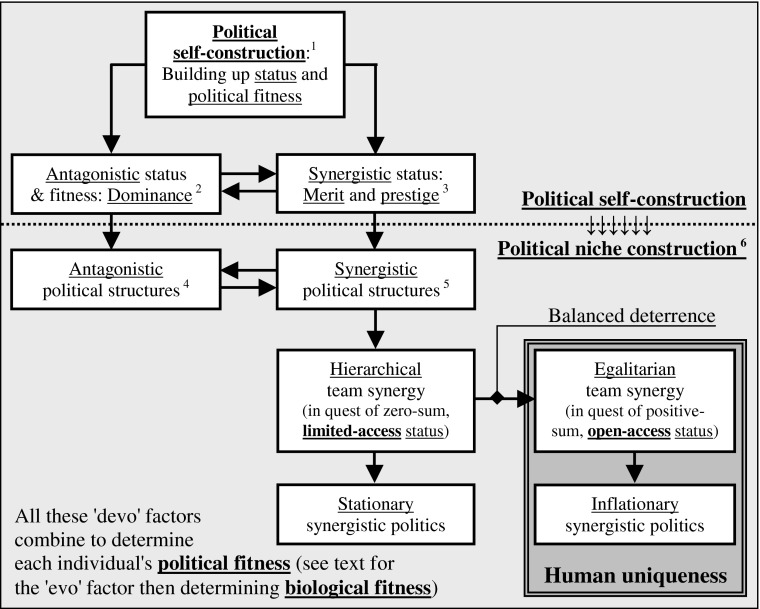
From political fitness to biological fitness: How does *political self-construction* in antagonistic and synergistic competition shape sociality? (1) In line with the principle of methodological individualism, this diagram emphasizes the single individual process behind all sociopolitical constructions: the lifelong, piecemeal, trial-and-error process of *political self-construction*. In this view, social animals progressively build-up, optimize and advertise their political fitness all along their lifetime. *Antagonistic self-construction* is the most basic and most universal form of political self-construction in social animals, followed by different forms of micro synergy, sometimes developing into whole-group hierarchical team synergy. In early humans, the dyadic balance of deterrence afforded by handheld weapons neutralized within-group antagonistic competition, thus leading to an inflationary escalation of *synergistic self-construction* among antagonistic equals. (2) Individual *dominance* derives from “who gets what,” the main stake in antagonistic competition. (3) Individual *merit and prestige* derive from “who brings what to the common good,” the main stake in *synergistic competition*. (4) Examples of antagonistic political structures: hierarchies, dominance ranks, hierarchical teams. (5) Examples of synergistic political structures: (a) micro (dyadic and triadic) partnerships and friendships; (b) quorums, parties, majorities, whole-group teams, and the associated leadership structures. (6) “Niche construction” as an evolutionary factor was originally proposed by Odling-Smee et al. (1996) and Laland et al. (2000)

No wonder why, in contrast, the “devo” quest for dominance and limited-access prestige in social animals can only lead to stationary cultures and communications always constrained within the same recurring—thus evolutionarily stationary—antagonistic equilibriums (Fig. 2). But then, what about the “evo” arm of the process? In other words, how could an individual’s “devo” political fitness be turned into “evo” differential reproduction (biological fitness) in a realm of egalitarian team solidarity? In normal times, within such egalitarian groupings of many monogamous pairs (see below) one would expect very little differences in fecundity between participants, somewhat like what happens in monogamous animals. What’s more, and in contrast with animals, egalitarian early humans could count on their teammates’ help in times of individual hardship, a temporary parental incapacity because of sickness or injury for example (Gurven et al. 2000). Where could differential reproduction come from in such uniquely egalitarian settings?

Of course, a whole team’s synergistic fitness should influence its successes and failures in between-team competition, and the ensuing group-selective process could bear on the differential reproduction of all teammates, as repeatedly suggested since Darwin (1871). But there is a much more powerful, individually selective process at work here. Indeed, in one recurring kind of hardship the available help from teammates is necessarily limited, and thus predictably distributed very selectively according to each one’s cumulative merit. This is in times of *collective hardship*—famines or surprise attacks by enemy neighbors, for example—evolutionary bottlenecks, therefore, wherein each one’s political record can turn into differential survival and reproduction. The selective mechanism is strategic and subtle, and it is probably the most basic “evo” (ultimate) reason why the veil of ignorance over synergistic competition is so deeply ingrained within the human psyche. However, its examination is better delayed until the second half of this paper. First, with the actual theoretical outline in mind, we must examine the more proximate “devo” factors behind this veil of ignorance, the reasons why the uniquely synergistic dynamics at work in early humans could have remained so barely accessible to the declarative awareness of all participants, and this, throughout the millennia during which, as a result of the same mighty dynamics, these increasingly gifted debaters were putting ever more words on ever more features of their world. Why ignore the most strategic of these features, the subtleties of competitive merit-seeking among peers? To answer this question, the next section discusses a few other stark differences between the “devo” quest for dominance and that for merit.

## Lifting the Veil

The proposed further comparison between limited-access dominance and open-access merit first boils down to four new opposites: dominance is altogether imposed, one-dimensional, transitive, and symmetrical, whereas merit among antagonistic equals is the exact opposite since it is altogether granted, multidimensional, intransitive, and antisymmetrical. These opposites are now examined one by one, they all contribute to dominance’s straightforward assessment and advertisement, in contrast with the intricate subtleties and constraints veiling merit’s assessment and advertisement:

1. *Dimensionality:* Individual dominance (antagonistic fitness, a capacity to take and keep something against the will of a fellow group-mate) is essentially one-dimensional since, in animals, it ultimately rests on mere strength and fighting ability. In contrast, individual merit (synergistic fitness) is multidimensional since, especially among antagonistic equals, there is an infinite number of possible ways and means for an individual to acquire merit in the eyes of regular partners and teammates:
  - differential merit in different collective tasks—hunting, defense, foraging, childcare, etc.;
  - differential merit according to the different, often opposite requirements of different collective tasks—strength vs. skill, boldness vs. cautiousness, swiftness vs. precision, etc.;
  - differential merit according to the different preferences of different partners.

Note in passing the similarity between the multidimensionality of merit and that of intelligence.

2. *Transitivity:* Because of its one-dimensionality, dominance is also *transitive,* both properties making it very easy to assess and advertise. That is, among three contestants A, B, and C in antagonistic competition, if A > B and B > C, then A > C; hence the clear-cut linearity of antagonistic rank orders (A > B > C > D…). In contrast, because of its multidimensionality, merit can be very *intransitive* (Henrich and Gil-White 2001:170) and thus much more difficult to compare from one individual to another. Indeed, individual A can gather more merit than B in activity X (AX > BX), while B can gather more merit than A in activity Y (BY > AY), whereas B can equal A for activity Z (AZ = BZ) and so on, not to mention the intransitivity of the preferences of those who grant this merit. Differential merit therefore becomes very difficult to assess precisely, and in absence of language and gossip, it remains invisible for non-witnesses and outsiders.

Note however that if A > (B, C, D…) in many activities X, Y, Z… and in the eyes of many partners and teammates, A’s cumulated merit translates into higher synergistic influence, which in turn translates into some amounts of prestige and leadership. These latter cumulative forms of merit therefore appear as two forms of “synergistic dominance,” which must not be confused with their one-dimensional antagonistic counterpart, the reason why the term *dominance* is here reserved for antagonistic forms of political influence. However, while merit is invisible for non-witnesses and outsiders, in contrast, prestige and leadership share some of the *salience* of dominance, which makes them both easily visible even for outsiders. This salience will become extremely important in the transmission of language and culture. Indeed, *all humans start their political career as cultural outsiders*—that is, as naive political actors totally dependent on salient models of political fitness so as to guide the building up of their own political fitness all along their development. *Each generation will therefore tend to reproduce the most salient—thus already “proven”—cultural and political models of the previous generation.* Note also that, for beginners and outsiders, the numerous possible “what, where, when, how, and with whom” of the quest for open-access merit become as many possible occasions to make costly developmental mistakes, another good reason for them to stubbornly stick to the most salient and obviously successful political models, whatever those models are (see below, about the post-Neolithic transition from egalitarian to strikingly unegalitarian models).

3. *Symmetry:* Whatever the salience of leadership and prestige, dominance nonetheless remains superiorly salient because it is also *symmetrical.* Indeed, there is always an obvious tangible symmetry between an individual’s antagonistic rank and the same individual’s material holdings, an obvious symmetry between “who takes what’ and “what is taken”; hence the well-known effectiveness of RHP (Resource-Holding-Power) displays. In contrast, among antagonistic equals such as human hunter-gatherers, all material resources (food, shelter, etc.) are shared equally, while inequalities in merit acquired through daily differential contributions to the common good can be easily blurred, especially when merged within the joint contributions of many teammates. *The symmetry between an individual’s merit and his/her material holdings is therefore broken, even reversed,* since the less one takes for oneself, and the more one brings to the common good, the more one’s merit grows in the eyes of witnesses. This *antisymmetry* therefore becomes the rule of the ensuing “competitive altruism” (Barclay 2004; Hardy and Van Vugt 2006; Roberts 1998; Van Vugt et al. 2007) once such a game (who brings more to the common good) develops among antagonistically equal teammates, further increasing the intricacies associated with the multidimensional assessment of inequalities in synergistic fitness.

Again, note in passing how, in a realm of egalitarian team synergy, as in that of human intelligence, “inequalities” in merit often derive from “differences” in talents, leanings, and abilities, such multidimensional differences in personality (see Wolf et al. 2007 on the evolution of animal personalities) often improving the whole team’s synergistic capacity instead of lessening it. Yet again, while contrasted personalities can improve a team’s effectiveness, they can also foster its fission into diverging quorums.

4. *Forcibility:* Last but not least, whereas dominance is *imposed* upon subordinates, merit is freely granted (Henrich and Gil-White 2001) according to contributions to the common good, provided these contributions are witnessed and appreciated by fellow group-mates. In other words, the synergistic influence derived from differential merit among antagonistic equals is conditional on the recognition and appreciation of partners and teammates. Such a *political status therefore becomes a positional asset freely granted according to the likings of peers who are simultaneously competitors for the same open-access status and the same synergistic influence.* Since these competitors can easily change their minds if the least bit displeased, the acquisition, buildup, and advertisement of merit is necessarily exposed to extremely demanding constraints, most of them diametrically opposed to those of dominance. That is, like dominance, one’s differential merit must be won over that of competitors, but unlike dominance, *one’s merit must not overshadow one’s peers’ own merit on pain of upsetting them,* thus reducing one’s merit instead of raising it. Of course, the same holds for prestige, the most coveted form of cumulative merit.

Remarkably then, the simple fact that merit and prestige are granted and cannot be imposed in antagonistic equals ensures that *tactful modesty when building up and advertising one’s own merit becomes compulsory, a “declarative” restraint* all the more indicated considering the unavoidable entanglement of individual and collective merit in a realm of egalitarian synergy. In addition and for the same reasons, a double form of compassion (solidarity in both successes and failures) becomes equally compulsory: how indeed could anyone rejoice about one’s own success and merit if no one else has any reason to rejoice? Better wait for this success to be shared so as to rejoice in common—or even better, use one’s talents and resources to lessen others’ difficulties, thus further improving one’s merit. Not only is arrogance counterproductive in such circumstances, and not only are modesty, fairness, generosity, and compassion better indicated, but most importantly, *all such displays of apparent selflessness necessarily undergo a competitive escalation, in close step with that of altruism.* Remarkably then, despite a thorough exploration of human (antagonistic) self-deception, Robert Trivers (2011) could have missed its brightest, most important form: the modest and empathically synergistic one distinctive of egalitarian humans. However, lest this appear too good to be true, note how *all these powerful synergistic constraints are tightly team-specific and can rapidly reverse into genuine hostility and counter-compassion—passionate chauvinism or xenophobic scorn—after a single bitter political scission.* Indeed, a conspicuous detestation of the opponent team is often required of all participants in between-team competition, as in within-team displays of bellicose solidarity.

In sum, the stark differences between dominance and merit among equals suggest how, all along humans’ egalitarian past, synergistic competition and the “devo” quest for differential merit could have been concealed by competitive modesty, solidarity, and compassionate altruism in interior politics, while being totally “outshined” by their overt counterpart in exterior politics, the arrogant detestation of opponent parties and enemies. This would only add to the subtleties associated with individual merit’s assessment—multidimensionality, intransitivity, reversed symmetry, and subjectivity. But there are still more confusing “devo” factors. To see them, one must now clarify the crucial differences between dominance and the three different salient forms that prestige takes in different political settings. As already suggested, the salience of prestige (cumulative merit) can be easily confused with that of dominance, thus adding to the fog surrounding differential merit, especially in the hierarchies of the post-Neolithic era, those of modern meritocracies included.

## Prestige’s Three Contrasted Profiles

For egalitarian humans first, displays of dominance and prestige mingle in a confusing way in exterior politics. As mentioned above, synergistic displays of individual might and bravery against enemies can bring major improvements to a team’s security, thus deserving much merit in the eyes of teammates (interior politics), such prestige yet remaining purely antagonistic in the eyes of enemy neighbors (exterior politics). But this confounding effect is even stronger in a realm of hierarchical team synergy where, in both interior and exterior politics, the same synergistic displays of strength and stamina—in both collective hunting and collective defense, for example—provide reliable indications about an individual’s combined synergistic and antagonistic potential, the latter remaining always available to enforce the internal rank order when needed (Zahavi 1977, 1980). In this way, team-living animals (cooperative breeders) can advertise their own antagonistic capacity, and appraise that of their teammates, without necessarily confronting them directly—that is, without breaching within-team discipline and without overtly contesting the current rank order. This notion of prestige as a handy substitute for dominance has been repeatedly emphasized by Amotz Zahavi (1977, 1990, 1995; Zahavi and Zahavi 1997) and followers, thus pointing to the possible significance of prestige in animal cooperative breeders. However, two crucial points remain to be acknowledged: (1) the fact that, within hierarchical teams such as those of cooperative breeders, the quest for prestige is always restricted to each one according to one’s dominance, hence the proposed notion of a *“limited-access,” imposed form of prestige,* whereas (2) the evolution of humans’ unique prosocial capacities was driven by the quest for a purely *“open-access,” freely granted form of prestige,* freed from dominance and only possible among antagonistic equals. Now, these two points can be further clarified by combining them with the already discussed differences between dominance and merit, this leading to three kinds of prestige, only one exclusive to egalitarian humans:

1. the *low-profile, open-access compassionate prestige* of egalitarian *primus inter pares*
2. the *high-profile, limited-access authoritarian prestige* of autocrats and hierarchical leaders.
3. the *high-profile, bellicose prestige* of warriors and conquerors.

In this view, P2 and P3 are familiar features of hierarchical team synergy in both animals and humans, while P3 is also common in egalitarian humans’ exterior politics (between-team competition). P1 is also familiar, but curiously, its political significance seems less recognized even though it appears as the only human singularity. Consequently, this paper’s core proposition can be more precisely rephrased as follows: *the competitive quest for open-access merit and low-profile compassionate prestige in a realm of egalitarian team synergy is the single*—*necessarily veiled*—*politically selective process responsible for human uniqueness’s developmentally propelled evolution all along the Paleolithic era* (Fig. 2). Now, as a next step in this model’s exposition, the following briefly examines how the confounding factors between dominance, merit, and prestige would only strengthen all along human evolution’s three main eras, the Paleolithic, the Neolithic, and the post-Neolithic.

## Low-Profile Prestige’s Ups and Downs

Throughout the Paleolithic era—that is, within early humans’ egalitarian societies—synergistic competition and the quest for open-access merit and compassionate prestige (P1) would have been systematically downplayed by their best practitioners, thus remaining clouded behind an enduring veil of declarative self-denial, with the ensuing emphasis on collective rather than individual merit further adding to the blurring effect of the latter’s intransitive and antisymmetrical multidimensionality. Despite this cloudiness, *the salient and compassionate prestige of each group’s best synergists would effectively guide the self-development of all group members all along their political career, such spontaneous mentorship thus ensuring the steady transmission of each group’s most merit-worthy synergistic signals and cultural ways and means all along the Paleolithic.*

As a result, competitive modesty, solidarity and compassionate altruism would remain powerful yet derived (secondary) factors favoring the egalitarian team dynamics primarily triggered and sustained by balanced deterrence. Therefore, being veiled and always dependent on the primary factor of balanced deterrence, these derived factors would rapidly lose much of their political traction during the Neolithic era, with the fast return of overtly stratified societies triggered by balanced deterrence’s breakdown. Indeed, a new capacity to stockpile nonperishable food resources such as grain and cattle would allow some Neolithic warrior elites to buy themselves personal militias, thus putting an end to primeval egalitarianism. The salience of these nascent aristocracies’ antagonistic manners, notably their uniquely conspicuous displays of material riches, would henceforth confuse all human minds, these being strongly bent on the egalitarian emulation of their most impressive elites—originally compassionate *primus inter pares,* now obliviously turning into authoritarian protection-racketeers.

Nonetheless, thanks to their deep evolutionary roots, competitive modesty, compassionate solidarity, and low-profile prestige (P1) would persist as powerful egalitarian and synergistic propensities in all modern humans. But since these deep-seated cravings strengthened the veil of ignorance over egalitarian synergy’s dynamics, they would often remain politically defenseless in front of the high-profile prestige (P2 & P3) of authoritarian ruling classes, whose combined dominance and prestige could be backed-up by ever-escalating antagonistic means of political power. The ensuing heavy fog of limited awareness about humans’ brighter egalitarian heritage would only thicken with the advent of the uniquely diverse and stratified cosmopolitan societies of post-Neolithic empires and civilizations—contemporary winner-take-all meritocracies included (Frank 1999; Frank and Cook 1996). Last but not least, the strong team-specificity of humans’ primeval political counter-compassion would allow some human groups to conquer and ruthlessly exploit other human groups, without the exploiters ever doubting their own “higher” merit-worthiness. On the contrary, such between-group differences in mere antagonistic success would be recurrently considered as “obvious” proofs of higher individual and collective merit-worthiness, and thus, higher individual and collective intelligence (Diamond 1997).

This brief historical outline therefore suggests how, *for the past ten thousand years (since the onset of the Neolithic era and throughout recorded history) and without any clear awareness of egalitarian synergy’s brighter dynamics, humans have been obliviously envying and emulating political elites who most often advertised*—*and still advertise*—*the silliest, most materially ostentatious, and thus most ill-adaptively attractive forms of political status.* No wonder why, then, the utmost contrasts between the different kinds of political greed for different kinds of political status—dominance, merit, P1, P2, and P3—are still mostly overlooked in both human behavioral ecology and political economics. An insatiable greed for status can be a very powerful passion indeed, driving individuals’ development and shaping animal and human societies in all sorts of critical ways. But obviously, all forms of greed are not equal; hence the importance of separating the light from the darkness in such strategic matters, especially in contemporary meritocratic economies (Frank 1999, 2007). For such darker political aspects of human nature to be eventually overcome, their brighter counterparts must first be properly understood. To this end, I turn to the abundant empirical evidence on the universality of human hunter-gatherers’ egalitarianism, and then to the evolutionary model proposed here to explain it. The whole is preceded by a brief review of John Rawls’s (1971) famous *top-down, virtual* veil of ignorance, as opposed to the bottom-up, adapted veil of ignorance discussed thus far.

## From Virtual to Primeval Equality

Instead of an evolutionary outcome, Rawls’s (1971) veil of ignorance is a thought experiment where a few virtual constituents are brought together so as to devise the social contract of a just society. For their decisions therein to be unbiased, the constituents must be ignorant of their future endowments, both positive and negative, thus ensuring the fairness of their future society’s institutions. Indeed, if no one knows in advance how one will end up in this society, as fit or sick, or as master or slave, for example, everyone will “logically” settle for institutions caring for the sick and barring both masters and slaves. Such a veil of ignorance on the future therefore creates hypothetical conditions from which normal humans can derive the principles of a fair, egalitarian society. Yet, after almost four decades of fruitful developments in political philosophy with this virtual approach, Amartya Sen (2009) recently emphasized its most important weakness: it is of great value to derive ideal principles, but without any means to enforce these ideals, their practical utility remains questionable. So, instead of pursuing such barely accessible ideals, Sen proposes a more realistic approach focused on the correction of the actual world’s most flagrant and still countless injustices.

However, such a step back away from the ideal can appear equally questionable in view of the abundant anthropological evidence on human egalitarianism. Indeed, after more than 300 years of “ethnographical” reports from all continents and latitudes, egalitarianism—at least among men; the trickier condition of women is discussed below—is one of the best documented human universals, typical of all hunter-gatherer societies (Knauft 1991; reviewed in Boehm 1993, 1997, 1999, 2012; Erdal and Whiten 1994, 1996:141–146). Although this “pristine” egalitarianism is often underrated as a mere consequence of the material poverty of hunter-gatherer societies as compared with the affluence of post-Neolithic stratified societies (see, e.g., Borgerhoff Mulder et al. 2009), such an underrating is most probably incorrect since our closest relatives, the chimpanzees, build up materially much poorer yet hardly egalitarian societies (Goodall 1986). Human egalitarianism is also often construed as a mere insurance against the high variance of hunting returns (see, e.g., de Waal 1996). However, chimpanzees also hunt, and they do share their kills, although very selectively, not to mention the numerous social carnivores who share their common catches but in a strictly biased, limited-access manner. This is in great contrast with the food sharing of human hunter-gatherers, which is always strictly egalitarian. Indeed, after reviewing much of the available qualitative and quantitative evidence, Erdal and Whiten (1996:142) conclude that not only do all modern hunter-gatherers share food, but “they do not share only with kin; they do not share only with those who reciprocate; they share out what they have according to need, even when food is scarce.”

And there is still more to humans’ documented primeval egalitarianism: it is also a unique instance of highly diversified whole-group cooperation, managed through consensual decision-making and without any hierarchical order, only an informal leadership without any coercive power (Erdal and Whiten 1996:144–145) but that of deliberative persuasion. Again, this is in great contrast with chimpanzee males’ dominance and sexual privileges (Goodall 1986; de Waal 1982). Once combined with the fact that hunter-gatherer societies are always composed of many monogamous pairs simultaneously raising numerous highly vulnerable families in close proximity (another stark contrast with chimpanzee males’ paternal irresponsibility, as with other cooperative breeders’ highly biased reproductive privileges), human hunter-gatherers’ universal pattern of closely knit, highly talkative, whole-group collective action in egalitarian solidarity calls for an explanation. The balance of deterrence afforded by handheld weapons is the single cultural trigger proposed here to explain the origin of this evolutionary singularity, with all its intricate mix of darker and brighter consequences. Balanced deterrence is only a trigger, but it might be the sole trigger allowing upright-walking primates to cross the uniquely demanding threshold separating hierarchical from egalitarian—thus talkative—team synergy.

The following therefore proposes a plausible model of humankind’s origins, the transition from pre-human chimpanzeelike hierarchical teams up to early humans’ egalitarian teams, the qualifier “chimpanzeelike” emphasizing the fact that modern common chimpanzees (*Pan troglodytes*) currently provide the best surviving models of protohuman males (Foley 1989; Ghiglieri 1987; Rodseth et al. 1991; Wrangham 1987)—a model of bonobolike protohuman females is examined below. A most paradoxical tale thus follows, where a simple cultural innovation, balanced deterrence thanks to handheld “technological” weapons (Chapais 2008),^3^ rapidly pushes some of the darkest antagonistic propensities of these chimpanzeelike males, their violence and machismo (Wrangham and Peterson 1996), up to an almost total neutralization and takeover by pure open-access synergistic competition in interior politics. Once imposed on all males—even the strongest ones—all along their development, such a competition would bear unprecedented devo-evo consequences for all males, females, and offspring, notably by throwing them all on the same runaway quest for open-access, freely granted merit, and this, under the same compulsory veil of compassionate modesty and generosity.

## Balanced Deterrence

The “discovery” of balanced deterrence stemmed from one single behavior change: our savanna-dwelling early male ancestors (most probably *Homo erectus* as explained below) eventually losing the powerful chimpanzeelike political inhibitions that prevented them from using handheld weapons (clubs, sticks, and stones) to injure fellow teammates. Why such political inhibitions at first? Because, for teamed-up philopatric males specialized in the defense and expansion of a common territory so as to attract and retain exogamic females, *the stringent requirements of team solidarity in exterior politics (between-team antagonistic competition) select for strictly inhibited, bloodless and weaponless combats in interior politics (within-team antagonistic competition).* The modern common chimpanzee (*Pan troglodytes*) offers a good illustration of such deep-seated political inhibitions (Boesch and Boesch-Achermann 2000; de Waal 1982; Goodall 1986; Nishida 1990; Wilson and Wrangham 2003) among mutually dependent obligate teammates. Chimpanzee males are indeed at the same time highly xenophobic, bellicose territory conquerors and proficient tool users. Remarkably, their technical culture is clearly overspecialized in subsistence technology (Whiten et al. 1999) and completely lacks fighting tools. For instance, chimpanzees carefully choose stones for cracking nuts, but they would never crack the skull of an opponent. Not that they are peaceful; quite the contrary: chimpanzee males can be very aggressive in within-team competition, and they are also extremely violent, determined, and uninhibited in between-team hostilities (Mitani et al. 2010). Yet, they remain essentially weaponless, bare-handed fighters in within-team conflicts, and they are also very ineffective, weaponless, and bare-handed bullies with captured neighbors (Goodall 1986; Wrangham 1999; Mitani et al. 2010). Their arboreal chases and escapes also impede weapon-carrying and weapon use. Overall, even if most of their antagonistic displays seemingly aim at inspiring terror, they remain as “bloodless” as possible in interior politics and the exact opposite in exterior politics.

Therefore, among already bipedal and similarly inhibited chimpanzeelike early humans, routine weapon-carrying and weapon use probably first developed for personal protection purposes because of the savanna’s scarcer arboreal refuges and much more numerous large predators. Then, once accustomed to routine weapon use against large predators (Kortlandt 1980),^4^ an essentially technical achievement affordable to both males and females, our male ancestors probably started using their handheld weapons in exterior politics (for territory defense and expansion so as to attract more females), thus becoming increasingly effective and politically uninhibited when terrorizing neighbors. Finally, once familiar with such uninhibited weapon use in exterior politics, the new fighting habit could spill over into the realm of interior antagonistic politics where such perilous experiments were previously impossible. It takes the repeated experience of weapon use in the synergistic solidarity of exterior politics (between-team terror), with the shared benefits of a more secure common territory to attract more females, to relax and eventually overcome the powerful inhibitions otherwise forbidding such experimentations in interior politics (within-team terror). But then, such a simple yet strategic cultural innovation—again *within a species of philopatric males already specialized in chimpanzeelike synergistic solidarity in exterior politics*—would bear numerous consequences, both technical and political, which rapidly combined into a whole new way of life with a clear paleontological signature.

Technically, in a brachiator’s hands, the kinetic power of a club’s blow or of a stick’s stab can be tremendous. As a fossilizable result, fighting with clubs should have selected for strengthened cranial bones, and Wolpoff (1980:177–178) reports such a thickening of the cranial vault for early *Homo erectus*, with brow ridges virtually doubled in size compared with earlier species, and with many reported healed scars, usually interpreted as hunting wounds. But political scars are also likely, and indeed, while suspecting ritualized head-bashing contests among males, Boaz and Ciochon (2004:78–88) emphasize the overtly defensive character of *Homo erectus*’ skull, its remarkable thickness being further reinforced by four equally remarkable interconnected bony bulges (torus occipitalis, t. angularis, t. supraorbitalis, and crista sagittalis), none of which needed as a muscle attachment ridge. This heavily armored skull makes *Homo erectus* a likely candidate as the first effective master of technological terror among hominids.

The evolution of *Homo erectus*’ armored skull necessarily took some time. But from the onset, it was much easier and faster to lose inhibitions than to evolve bodily and behavioral parries against primeval yet diversified handheld weapons. Moreover, since weapon use is a cultural habit, it could spread rapidly to all rungs of the preexisting antagonistic ladder. As a result, even lower-ranking males could now impose dreadful blows on their strongest competitors, especially in surprise attacks, the ensuing evenly distributed capacity to inflict terrorizing wounds leading to a genuine balance of deterrence in within-team antagonistic competition. Two features of this kind of political equilibrium must be underlined:

1. Balanced terror between individuals of unequal force results from the salient mutilating and incapacitating power of hand-accelerated weapons.
2. Such a balance of terror cannot evolve through ordinary genetic selection. Indeed, *bodily weapons (horns, teeth, claws, muscles,* etc.*) evolve slowly, in step with behavioral defenses and bodily armors,* the ensuing deterrence power being always dependent on age, weight and experience. As a result, the smallest differences in overall antagonistic capacity most often allow a profitable antagonistic order to be established, however short-lived it may be.^5^

The contrast between bodily and cultural weaponry is therefore striking: a sharp stick in the belly, or a club’s blow on the skull, necessarily have dreadful effects, whatever the weight or size of the attacker. In combination with related greatly increased risks in between-team clashes, the ensuing within-team balanced terror would thus favor a totally unprecedented kind of team-wise political order: within-team antagonistic equality in males, with uniquely increased needs for team solidarity (synergistic political stability), arising from two complementary factors. First, among uninhibited users of handheld weapons, *personal security comes to depend much less on personal strength than on a capacity to make reliable friends* since such primitive weapons only ensure a one-to-one equality, which breaks down at two against one; hence the necessity of competitive recruitment and defensive quorum formation. But second, within-team antagonistic equality in males would greatly improve the reproductive potential of all but the strongest male dominants, who would now have to share mating opportunities as never before. This improved sexual and political equality in males would thus foster monogamy and paternal investment (see below), which would equally benefit all females and infants by ensuring them an equal access to all the material requirements of daily survival—notably food, shelter, and protection. But this would then boost demographic expansion, within-team and between-team antagonistic competition, and the associated need for long-term solidarity with sufficient teammates. The transformation of our ancestors’ mating behavior is examined first.

## Bonobolike Females

In contrast with males, protohuman females were probably more bonobolike than chimpanzeelike. Indeed, the modern bonobos (*Pan paniscus*) provide a better model to understand the improved solidarity of these females (Wrangham 1993), life in the savanna imposing on them much fewer daily dispersions (fissions-fusions) than those of forest-dwelling chimpanzee females (Pruetz and Bertolani 2009); hence the improved familiarity and possible solidarity of protohuman females. However, as proposed by O’Connell et al. (1999, 2002), protohuman females probably had a much harder time provisioning their children than chimpanzee and bonobo females do because (1) the scavenged meat and marrow provided by males was most probably too scant and unpredictable (but see Bunn and Gurtov 2014) and (2) the savanna’s abundant and nourishing tubers were much harder to unearth and process, as compared with the simple picking of fruits that chimpanzee offspring can rapidly manage. As a result, protohuman females had a heavy parental burden, and they probably remained dominated by their philopatric males, specialists of “high-profile” terror politics, zero paternal investment, and meat and marrow provisioning as mere self-promotional displays.

How would all this change once antagonistic equality became firmly established among males? Within-team balanced deterrence among males would first offer lower-ranking males new, unprecedented opportunities for mate choice and mate guarding within the stable consortships normally sought after by lower-ranking chimpanzee males (Goodall 1986). Because such consortships would no longer be disrupted by higher-ranking males, pair-bonding would begin to be favored from the bottom of the political ladder up. Conversely, from the top down the same ladder, once the quest for modest and compassionate merit became firmly established as the rule of the political game, the more coveted and merit-worthy males and females would tend to pair together and avoid overt extra-pair solicitations so as not to overshadow their lesser competitors, thus avoiding demerit and the risk of quorum punishment. As a result, from both ends of the political ladder, all males and females would tend to pair with sexual partners of comparable merit and synergistic fitness, thus favoring monogamy, as predicted by the famous “stable matching algorithm” of Gale and Shapley (1962). This in turn would rapidly select for paternal investment, family protection, concealed ovulation, and reduced sexual dimorphism in sexually bonded pairs. Indeed, improved possibilities for all human males to claim and defend well-identified females would foster paternal investment, but it would also make their no-longer-promiscuous females uniquely vulnerable to the sexual and infanticidal harassment by other males (Hrdy 1977; Smuts and Smuts 1993), thus rapidly selecting for concealed ovulation. That is, monogamy would relieve human females from their ancestor’s chimpanzee/bononbolike defenses based on advertised ovulation and sexual promiscuity for paternity confusion and infanticide avoidance (van Schaik and Janson 2000; Wrangham 1993).^6^ In contrast, concealed ovulation and the benefits of solid parental pairs would drive human females’ sexual receptivity toward its exuberance, continuity, and pair-wise selectivity. The ensuing sturdier, sexually bonded pairs with helpful and dependable fathers would greatly improve the reproductive output of early human mothers, through earlier weaning, more numerous pregnancies and simultaneously dependent children, but also thanks to the egalitarian solidarity of other mothers (Hrdy 2009) and grandmothers (O’Connell et al. 1999). This in turn would fuel the unique demo-geographical expansion of *Homo erectus*.

This trend away from chimpanzeelike male irresponsibility would also bolster the need for political stability. Indeed, because of human infants’ vulnerability and uniquely prolonged dependency, long-term family security among diverging quorums and factions would require uniquely prolonged and reliable associations within sufficiently large teams. Dispersion would thus be often precluded, and the ensuing increased number of reproductive pairs within each team would inevitably increase the risks of unmanageable conflicts, with related heightened risks of political instability and political scissions. This is where balanced deterrence produces its most paradoxical effects since, while precluding dispersion and fueling an unprecedented need for political stability, it also fuels political instability and political scissions, neutralizing the foremost factor of political stability in all other team-living animals (notably cooperative breeders): a stable antagonistic rank order with the related policing services normally provided by all teammates according to their dominance rank (Clutton-Brock and Parker 1995). In such peculiar circumstances, another strategic life-insurance stake fuels the escalation of both egalitarian solidarity and synergistic competition, the quest for differential merit remaining a daily necessity so as to eventually win preferential assistance in times of *collective hardship.* This latter challenge was briefly evoked above (“Political Selection’s Devo-Evo Mechanics”), and it can now be discussed in more detail. Again, it appears as a key “evo” factor responsible for the biological fitness (differential reproduction) which stemmed from modest and compassionate merit all along humans’ egalitarian past.

## Runaway Solidarity

First, it should be recalled that, for our egalitarian ancestors with uniquely prolonged parental responsibilities, one obvious benefit of team solidarity and enlightened teammate choice becomes the protection that they provide against individual misfortunes of all sorts (sicknesses, disabling injuries, etc.), any one of which can jeopardize many years of parental investment. This is in great contrast with what happens in the hierarchical teams of other cooperative breeders, where non-breeding helpers care for a dominant pair’s seasonal progeny. Among early humans, many monogamous pairs were simultaneously raising vulnerable infants, any one of which could be lost after just a few days of parental incapacity during the many years of offspring dependency. Such a unique vulnerability combines with the mutilation risks brought about by weapon use in between-team conflicts. Since anyone can be stricken in such circumstances, and since any teammate can help, a tightly bonded team becomes a mutual insurance arrangement to which all teammates must subscribe in order to get the necessary assistance when required.

But there is a most peculiar twist to such an insurance arrangement among a small number of egalitarian “subscribers.” Indeed, not only is a good political record necessary to be assisted in case of individual misfortune (Gurven et al. 2000), but, as mentioned above, many instances of individual bad luck happen in circumstances of collective hardship in which (1) the progenies of many reproductive pairs are simultaneously at risk and, consequently, (2) the mutual fund’s capacity to protect its subscribers against the ongoing risks is overwhelmed by the number of claimants. Thus, with many teammates simultaneously in need of assistance, the ability of a victim and her progeny to be assisted will not depend on the absolute amount of “premiums” paid into the mutual fund. It will rather depend on the *difference* between the total premiums paid by different victims (the difference between their previously accumulated merit), with the most-esteemed victims tending to gather a greater part of the limited available help. For example, in the heat of a surprise attack by enemy neighbors, with many casualties all around, one will tend to help one’s alpha best friend before one’s gamma best friend.

This is therefore another critical reason why the “devo” quest for differential merit would remain a necessity for all humans whatever their sex and seniority, with the smallest differences in accumulated merit (political fitness) having potentially critical consequences on survival and differential reproduction (biological fitness), especially in times of evolutionary bottlenecks. And again, because all surpluses in merit had to be downplayed in order to benefit their owners, this political imperative would only strengthen the competition for fairness and generosity behind the veil of competitive modesty and compassion. Paradoxically then, despite its inherent political instability, and beyond the inflation of synergistic communications that it fosters, another major consequence of our ancestors’ dread-laden egalitarianism would have been a most cheerful expansion of a competitive quest to make friends with as many teammates as possible, by sharing their joys and sorrows and by playing on their preferences, notably for competent teamwork and dependable solidarity in hardships. Such a runaway quest would select for uniquely powerful capacities and cravings for empathy, fairness, and compassion. In sum, balanced terror would transform early humans into champions of friendship and virtuosos of empathy and solidarity, a rare evolutionary quirk.

## *Ecce Homo*

This model explains many contradictory aspects of hunter-gatherers’ sociality that have always been difficult to reconcile, notably (1) the most cheerful aspects of primitive humans’ egalitarianism, which have fascinated the New World’s early European discoverers and many contemporary cultural anthropologists, and (2) the much darker aspects pertaining to warfare with endless cycles of collective revenge, between-group terror, ritual torture, and cannibalism (Chagnon 1988; Keeley 1996; LeBlanc and Register 2003; Meggitt 1977; Staden 1557, among others). The century-old myth of the noble savage may not be a complete myth after all, with nobility’s functional roots being veiled in interior politics and overshadowed by their darker counterparts in exterior politics. Among these darker aspects, antagonistic equality among men also explains the extremely variable status of women in hunter-gatherer societies, a status which can vary from near equality to the worst forms of subordination (Hayden et al. 1986). Indeed, balanced deterrence among monogamous men would result in antagonistic equality for all of a group’s families—that is, equal access to all of daily survival’s material requirements (food, shelter, etc.) for all men, women, and children. But despite this broad material equality, the antagonistic subordination of women would persist for two reasons: (1) because of the recurring and prolonged periods of lesser political leeway associated with pregnancies, breast feeding, child care, and other highly demanding, family-focused maternal duties (a heavily biased mammalian burden which would only lessen thanks to modern contraception) but also, and most importantly, (2) because of *the uniquely demanding political challenges resulting from early human females’ chimpanzee/bonobolike exogamy* (Ghiglieri 1987; Wrangham 1987). Indeed, exogamy forces all adults of the migrant sex (here, females) to start their political career—their political self-construction—afresh within the new competitive environment of their group of adoption, an extremely challenging endeavor demanding *an added amount of low-profile, synergistic knack and tact, together with an added amount of talkative competence.* Therefore, the antagonistic subordination of women would not reduce the pressures of synergistic competition upon them—quite the contrary. It would make political self-construction and the quest for merit separate intrasexual arenas, with each gender developing specialized areas of expertise (Murdock 1969). Then, because of the pressures and necessities of mate choice and parental pair formation, each gender’s intrasexual synergistic contests would necessarily unfold under the close scrutiny of the opposite sex, which would result in a most demanding twofold form of whole-group synergistic competition, with everyone trying to excel in one’s intrasexual sphere of competence, thus simultaneously maximizing one’s chances of success in the realm of intersexual partner choice. Since excellence is most often measured in contributions to the common good, the following briefly reviews how such team-wide synergistic competition powerfully fuels the transmission and expansion of the best of each group’s culture.

## Egalitarian Culture’s Veiled Magic

Hunter-gatherers’ egalitarianism thus becomes a remarkable political system in which the elite is normally composed of the most esteemed and admired individuals, those who bring the most to the common good while taking less for themselves. In so doing, this open-access elite of mentors and *primus inter pares* exerts more synergistic influence, and it piles up more merit and prestige. But by producing and contributing more collective benefits, this synergistic elite also advertises the best practices in many domains of expertise—the most effective and appreciated technical and political behaviors. Translated into contemporary terms, those of modern liberal democracies, these meritocratic elites do not claim for themselves any material privilege—an imperative self-restraint in a realm of competitive low-profile prestige and dignity. As a reward for their higher contribution to the common good, *they are instead given more influence on technical and political quorum formation.* That is, they are granted a greater influence on different aspects of collective action and collective deliberations—*and therefore a greater capacity to contribute to the common good, and thus pile up more merit and low-profile compassionate prestige.* Of course, the advertisement of such elite practices requires individual qualities such as competence, know-how, generosity, effectiveness, trustworthiness, modesty, compassion, etc., synergistic qualities that can build up to different levels from one individual to the next. But since these qualities are purely informational riches, they can all be shared without impoverishing their owners. To the contrary, such qualities must be shared and displayed in order to be turned into individual assets such as merit, thus favoring their imitation by younger or lesser competitors. As a result, the more these qualities are displayed by their best practitioners, the more they can be imitated by followers and admirers. This uniquely synergistic magic has two important consequences.

First, it always levels the field of synergistic competition since, as emphasized above, to preserve their leadership and prestige, elite competitors must always be both modest about their expertise and generous in sharing it, thus allowing less-advanced followers to catch up. But second, as a result of this leveled competitive field, the quest for synergistic influence through *the advertisement of such elite synergistic expertise powerfully sustains the cross-generational persistence and expansion of this expertise*, with everyone emulating the most appreciated performers to the benefit of all. In fact, synergistic expertise needs to be shared, exploited, and thus advertised and emulated so as to persist in its adopters’ behavioral repertoire through the generations. And indeed, thanks to its informational nature, this synergistic cultural wealth can be shared indefinitely without risk of depletion: the more it is shared, the more it persists and expands, and the more it empowers its egalitarian sharers, both individually and collectively. Yet of course, nothing guarantees the soundness of the adopted behaviors: early humans unknowingly made all sorts of costly mistakes—notably, overexploiting their ecological environment and its megafauna (Barnosky et al. 2004; Martin and Klein 1984) or waging costly battles between rival quorums. But despite these unavoidable shortcomings of synergistic politics, the faithful transmission of egalitarian culture did guarantee that any expertise contributing to a group’s common good—rhetorical expertise, notably—would be faithfully transmitted down the generations, the ensuing cumulative process of political niche construction being thus propelled and sustained by the quest for open-access merit or, more precisely, by the simple trial-and-error “devo” process of “competitive synergistic self-construction among antagonistic equals” (Fig. 2).

**Fig. 3:**
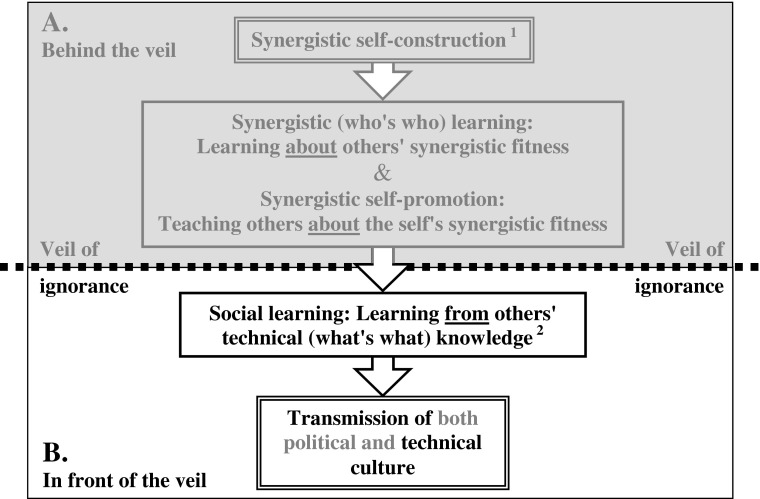
Upstream from mere social learning: An expanded model of cultural transmission through competitive *synergistic self-construction*. (1) This model applies to both *antagonistic* and *synergistic self-construction*. However, antagonistic self-construction is hardly veiled, even though the associated, political forms of who’s who learning and teaching seem more neglected. (2) “Technical (what’s what) knowledge” essentially refers to the ecological environment, that is: (a) inanimate things (plants, shelters, migration routes, etc.); (b) animals without an individual identity (prey, predators, ecological competitors, etc.). By contrast, *political (who’s who) knowledge* refers to familiar group-mates with an individual identity, and therefore knowledge about these individuals’ political fitness, both antagonistic and synergistic

Of course, synergistic self-construction requires a lot of social learning (learning from others), as emphasized by all current models of culture transmission (see, as a telling example, Boyd et al. 2011). However, in the realm of political synergy, such an essentially technical form of social learning appears as a tactical means to a much broader, all-encompassing end, that of political self-construction (political fitness-building). This latter end is all-encompassing because, beyond usual social learning (learning from others), it involves two other informational tasks of utmost significance: *synergistic who’s who learning* (learning about others’ synergistic fitness) and *synergistic self-promotion* (teaching others about the self’s synergistic fitness). In contrast to antagonistic fitness, whose salience and symmetrical one-dimensionality makes it easy to assess, the intransitive antisymmetrical multidimensionality of synergistic fitness makes it much harder to assess. Every dimension of merit must be witnessed in order to be appreciated, and any single behavior can reveal precious details about a partner-competitor’s synergistic strengths and weaknesses in one or another domain; hence the permanent necessity of vigilant synergistic who’s who learning and synergistic self-promotion (who’s who teaching), upstream from mere technical (what’s what) learning. Remarkably, then, the fact that most current models ignore these crucial political requirements of culture’s transmission further supports the idea that a now-ill-adapted veil of ignorance still conceals the magical dynamics of competitive synergistic self-construction among peers (Fig. 3).

Incidentally, note also how the same veil of ignorance explains another puzzling aspect of behavioral theories, the fact that cooperation is most often conceived as the opposite of unqualified “competition.” In contrast, the distinction proposed here between the two opposite forms—antagonistic and synergistic competition—clearly emphasizes at least three crucial facts:

1. that cooperation is far from always devoid of competition in social animals (see, for example, Roberts 1998; Zahavi 1977), especially when many would-be partners of variable effectiveness or trustworthiness are available (Noë and Hammerstein 1994); and therefore
2. that competition is far from always exclusively antagonistic or zero-sum; and finally
3. that humans are by far Nature’s most gifted specialists of a uniquely egalitarian and communicatively demanding form of team synergy—hence the necessity to lift the veil of ignorance over this kind of political synergy to understand human uniqueness.

## The Magic of Informational Commons

In conclusion, one crucial aspect of the human condition deserves final emphasis in light of the often-evoked distinction between material and immaterial/informational resources and constructions. In this view, humans’ uniqueness stems from their capacity to build up and share huge informational megastructures, those of language and culture, which are intangible pyramids of meaning cumulatively built up by thousands of generations of fellow humans. Again, because of their immaterial nature, these common informational riches can be shared indefinitely without risk of depletion. And this is in great contrast to all material riches, which should be exploited sparingly and efficiently so as to make sure everyone gets a fair and sustainable share. The fact that contemporary humans still cannot reach a sturdy consensus on the perils of *material greed* within limited material commons (Meadows et al. 1972, 2004) appears somewhat paradoxical, to say the least, and again it points to the veil of ignorance over the other contrasted kind of *immaterial greed* responsible for humans’ huge informational commons (language, knowledge, and culture). These informational commons are invaluable public goods whose cumulative construction through millennia is still barely clarified by science. Yet, if the “egalitarian hypothesis” of human uniqueness outlined here is sound, *the steady inflation of these common informational goods during more than 1.5 M yrs was driven by an insatiable greed for purely individual, immaterial (who’s who) assets such as merit and prestige, among people sharing all their material riches equally*. As a result, because of its intransitive antisymmetrical multidimensionality, and because it had to be downplayed, this insatiable greed for individual merit and prestige could have also concealed its most cheerful collective outcome, the simple magic behind the expansion of humanity’s immaterial commons. In other words, the now-ill-adapted veil of ignorance over synergistic self-construction among equals would have hidden the genuinely magical process of informational niche construction (Laland et al. 2000; Odling-Smee et al. 1996) behind humanity’s most precious assets, its magnificent, infinitely sharable and expandable informational commons.

In this view, humanity’s common stock of volatile yet infinitely sharable knowledge clearly becomes the only world in which unlimited growth is possible, the only market economy in which insatiable greed and boundless self-enrichment are both compatible with the common good and achievable for all participants, the only ecological niche whose unremitting exploitation is absolutely necessary for its own preservation and development. It is also a collective masterwork bequeathed by thousands of generations of clever fellow humans, unaware virtuosos of egalitarian solidarity. A collective masterwork, moreover, whose enhanced sharing and further expansion in the form of science and technology is now absolutely necessary to build up a more enlightened, more sustainable future for humanity’s overexploited material common, planet Earth with its endangered biosphere and atmosphere. In contrast to the optimism of pre-Darwinian science and the Age of Enlightenment, post-Darwinian science has most often been either very pessimistic about human nature (Dawkins 1976; Trivers 2011) or tragically misguided by the numerous political and economic guises of so-called Social Darwinism. The present paper suggests how humans’ synergistic blindness could have contributed to such pessimism and misconceptions. Could it be that an improved Darwinian understanding of humans’ unique synergistic and egalitarian propensities might finally allow the fulfillment of a longstanding humanistic dream, that of whole-humankind egalitarian synergy?

## Footnotes

1 Quorum: the number of members of a group required to carry out a collective action or synergistic activity.

2 See North et al. (2009) for the notion of “open-access” political power and competition, with numerous illustrations of their utmost significance all along human (post-Neolithic) history.

3 The present model agrees with Chapais (2008:176–177) on the deterrence power of “technological” weapons and the ensuing momentous consequences of pair-bonding on early humans’ sociality. However, the present model diverges markedly on the preferred primate model for protohuman societies. This difference is discussed farther below.

4 Kortlandt’s (1980) numerous defensive experiments with the savanna’s large predators show that these can be kept at a safe distance with a single hand-waved thorny branch, one of acacia for example. Indeed, if stuck into an eye or the muzzle, a single thorn can jeopardize survival even for the largest felines.

5 Note that because of dangerous bodily weapons, but also as a response to specially harsh pressures from predators or neighbors, some social animals spontaneously evolve milder hierarchies, sometimes nearing egalitarianism (Vehrencamp 1983). However, such a slow progress toward egalitarianism does not favor team synergy, for many interrelated reasons. First, whole-group team synergy can only emerge from an already stable rank order, with alpha dominants as leaders. But then, in order for it to develop further, teamwork must also be more productive than mere solitary work, with this greater productivity thus necessarily fueling both (1) the greater antagonistic competition associated with the division of teamwork’s greater collective outputs and (2) the greater synergistic competition associated with the greater requirements of teamwork’s management (leadership, followership, division of labor, etc.). Therefore, whatever the hierarchical order’s steepness from the onset, whole-group team synergy can only evolve toward even greater steepness for both the antagonistic and the synergistic political ladders. As a result, for team synergy to become egalitarian as it did in early humans, balanced deterrence must develop rapidly thanks to cultural weapons, and among animals already specialized in the mutual dependence and the disciplined forms of antagonistic and synergistic competition typical of hierarchical teams.

6 In a similar proposition about the evolutionary significance of “technological” weapons, Chapais (2008:176–177) argues that, instead of fostering monogamy, balanced deterrence among chimpanzeelike human males would instead increase scramble sexual competition (sperm competition) among them, thus leading to increased sexual promiscuity instead of pair-bonding. But this argument neglects the fact that *sexual promiscuity is not a male but a female strategy to confuse paternity and avoid infanticide.* Instead, Chapais models early human males as polygynous harem builders, united within multiharem structures similar to those of hamadryas baboons. However, such loosely associated males lack the political habit and benefits of team synergy and solidarity in exterior politics first necessary to develop weapon use against enemy neighbors, before importing this innovative behavior in the realm of interior politics, where such technical tinkering is otherwise hardly possible. Indeed, any trial of this type by lower-ranking males would be immediately thwarted by stronger dominants who themselves don’t need such technical expedients.
